# Supplementary material for: Recovery of Water Homeostasis in Adenine-Induced Kidney Disease Is Mediated by Increased AQP2 Membrane Targeting
Source: Int J Mol Sci. 2024 Mar 19;25(6):3447. doi: 10.3390/ijms25063447 (PMC10970147; doi:10.3390/ijms25063447)
Supplement: Supplementary file 1 [file ijms-25-03447-s001.zip › ijms-2872533-supplementary.pdf]

Table S1. Sequence of primers for quantitative reverse-transcriptase polymerase chain reaction.

Target gene	Direction	Sequence (5′ to 3′)
Gapdh (GAPDH)	Sense	TAAAGGGCATCCTGGGCTACACT
	Antisense	TTACTCCTTGGAGGCCATGTAGG
Fn1 (FN)	Sense	AATGGAAAAGGGGAATGGAC
	Antisense	CTCGGTTGTCCTTCTTGCTC
Col1a1 (COL1A1)	Sense	CAATGGTGAGACGTGGAAACC
	Antisense	ACAGTCCAGTTCTTCATTGCA
Il1b (IL-1β)	Sense	CAGGCAGGCAGTATCACTCA
	Antisense	TGTCCTCATCCTGGAAGGTC
Il6 (IL-6)	Sense	GATGCTACCAAACTGGATATAATC
	Antisense	GGTCCTTAGCCACTCCTTCTGTG
Ccl2 (MCP1)	Sense	CAAGAAGGAATGGGTCCAGA
	Antisense	GTGCTGAAGACCTTAGGGCA
Tgfb (TGF- β)	Sense	ACCGGAGAGCCCTGGATAC
	Antisense	AGGGTCCCAGACAGAAGTTG
Havcr1 (KIM-1)	Sense	CGGTACAACTTAAAGGGGCA
	Antisense	GACGTGTGGGAATCTCTGGT
Aqp1 (AQP1)	Sense	GTCCAGGACAACGTGAAGGT
	Antisense	ACACACTGGGCGATGATGTA
Aqp2 (AQP2)	Sense	CTTCCTTCGAGCTGCCTTC
	Antisense	CATTGTTGTGGAGAGCATTGAC
Aqp3 (AQP3)	Sense	TGTGTGTACTGGCCATCG TT
	Antisense	GTTGACGGCATAGCCAGAAT
Aqp4 (AQP4)	Sense	TGGTGTTCACTATTTTTGCC
	Antisense	GATCAAGTCTTCCGTCTCCA
Tnf (TNF-α)	Sense	AGGCTGCCCCGACTACGT
	Antisense	GACTTTCTCCTGGTATGAGATAGCAAA
Acta2 (α-SMA)	Sense	CTGACAGAGGCACCACTGAA
	Antisense	CATCTCCAGAGTCCAGCACA
Slc12a1 (NKCC2)	Sense	AGTGCGCCCAAAGTATTCCA
	Antisense	CAATGACATTCAGCTCGGCG
Slc9a3 (NHE3)	Sense	TCGGCAGGAGTACAAGCATC
	Antisense	AGCTTGGCCGCCTTCTTATT
mMrgA9 (Ade2R)	Sense	CACGTTCTCCTCACCCAACA
	Antisense	CCCAGATTGCAGCACACATG

Abbreviations: GAPDH, Glyceraldehyde 3-phosphate dehydrogenase; FN, Fibronectin; Col1a1, Collagen type 1 alpha 1; IL, Interleukin; MCP, Monocyte chemoattractant protein; TGF, Transforming growth factor; KIM, Kidney injury molecule; AQP, Aquaporin; TNF, Tumour necrosis factor; SMA, Smooth muscle actin; NKCC, Sodium-Potassium-Chloride Cotransporter; NHE, Sodium-hydrogen exchanger; Ade2R, Adenine receptor.

Table S2. Antibodies used for immunoblotting, immunohistochemistry, and immunofluorescence.

Target	Source	Dilution	Method	Manufacturer
**Primary antibodies**				
α-SMA	Mouse	1:500	Immunofluorescence	ab215368; Abcam
		1:250	Immunoblotting	M0851; DAKO
FN	Rabbit	1:50	Immunohistochemistry	ab2413; Abcam
		1:1000	Immunoblotting	
PDGFRβ	Rabbit	1:400	Immunofluorescence	ab32570; Abcam
KIM-1	Goat	1:400	Immunofluorescence	AF1817, R&D Systems
AQP1	Rabbit	1:200	Immunofluorescence	AB2219; Sigma-Aldrich
AQP2	Mouse	1:200	Immunofluorescence	sc-515770; Santa Cruz Biotechnology
AQP3	Rabbit	1:1000	Immunohistochemistry	AQP-003; Alamone
AQP4	Rabbit	1:4000	Immunohistochemistry	AQP-003; Alamone
NKCC2	Rabbit	1:2000	Immunofluorescence	38436S; Cell Signalling Technology
**Secondary antibodies**				
Rabbit	Donkey	1:500	Immunofluorescence	Alexa Fluor 647; Invitrogen
Goat	Donkey	1:500	Immunofluorescence	Alexa Fluor 680; Invitrogen
Mouse	Donkey	1:500	Immunofluorescence	Alexa Fluor 488; Invitrogen
Rabbit	Goat	1:4000	Immunoblotting	Alexa Fluor 447; Dako
Mouse	Goat	1:4000	Immunoblotting	Alexa Fluor 448; Dako

Abbreviations: SMA, Smooth muscle actin; AQP, Aquaporin; FN, Fibronectin; KIM, Kidney injury molecule; NKCC, Sodium-Potassium-Chloride Cotransporter; PDGFR, Platelet derived growth factor receptor.
